# Medical students’ perception towards E-learning during COVID 19 pandemic in a high burden developing country

**DOI:** 10.1186/s12909-021-02811-8

**Published:** 2021-07-10

**Authors:** Mohamed Daffalla-Awadalla Gismalla, Mohamed Soud Mohamed, Omaima Salah O Ibrahim, Moawia Mohammed Ali Elhassan, Mohamed NaserEldeen Mohamed

**Affiliations:** 1 Department of Surgery, Faculty of Medicine, University of Gezira, Medani, Sudan; 2 Department of Pediatrics and Child Health, Faculty of Medicine, University of Gezira, Medani, Sudan; 3 Department of Oncology, National Cancer Institute, University of Gezira, Medani, Sudan

**Keywords:** E-learning, Distance learning, Medical Education, Africa

## Abstract

**Background:**

In High-income countries, many academic institutions are using E-learning during COVID 19 Pandemic. However, in limited-resource countries, like Sudan, shifting towards E-learning requires many adjustments to be made to make sure the E-learning is held in a proper manner, as best as possible. This study was undertaken to assess medical students’ perception towards implementing E-learning during COVID 19 Pandemic and to highlight for E-learning implementation in Sudan as an example of a limited-resource setting.

**Methods:**

A cross-sectional survey was conducted between 10 and 25 of May 2020 among the undergraduate medical students at the Faculty of Medicine, University of Gezira, Sudan. The study used self-administered online-based questionnaire. E-mail and social media platforms such as Facebook and WhatsApp were utilized to disseminate the questionnaire.

**Results:**

The total numbers of 358 undergraduate medical students responded to the online survey questionnaire. The majority (87.7 %) of students agreed that the closure of the university is an essential decision to control the spread of the COVID-19 infection. Approximately two-thirds (64 %) of students perceived that E-learning is the best solution during COVID 19 lockdown. The level of medical students (Pre-clerkship and Clerkship) and place of residence had significant correlation (*p*-value < 0.05) with medical students opinion regards starting the E-learning. Internet bandwidth and connectivity limitation, unfamiliarity with E-learning system, technical support limitation and time flexibility in case of technical problems during online exams, and lack of face-to-face interaction were the factors considered by medical students to be against the E-learning implementation.

**Conclusions:**

Most medical students had a positive perception of E-learning. However, there are many challenges considered as an inhibitory factor for utilizing electronic technologies for medical education. We recommend that challenges of E-learning in our limited-resource setting should be systematically evaluated and that effective strategies should be developed to overcome their inhibitory effects.

## Background

In March 2020 World Health Organization (WHO) declared that Coronavirus disease 2019 (COVID-19) as a worldwide pandemic [1]. This pandemic is an unprecedented emergency that has affected all global industries, including education [2]. Moreover, as a result of social distancing, the most effective preventative strategy since the emergence of COVID-19 [3], medical education has been profoundly disturbed as it involves in-person didactic lectures and tutorials, clinical rotation exposure, laboratory experiences, observing and assisting relevant medical and surgical procedures [4, 5].

In this crisis, the need to encourage E-learning in the modern world of education becomes clear. E-learning platforms can be utilized to deliver lectures remotely at one’s convenience. Students can then log in at a scheduled time for discussions, which can be facilitated live using video and audio conferencing. E-learning has a positive effect if the student participates actively. In high-income countries, there is a good experience in E-learning learning in health professional education [6–8]. The situation is differing in low- and middle-income countries, few countries have some experiences in E-learning health professional education, mainly in postgraduates training [9, 10]. In Sudan, there is limited exposure to E-learning in high education. A limited number of Sudanese universities offer E-learning, such as the Open University of Sudan, which has 18 branches in Sudan and provide E-Learning through educational web sites, live broadcasting (video conferencing), educational discs, Electronic Library, TV channels, and educational radio. Sudan University of Science and Technology offers E-learning for a master’s degree in computer-integrated Education. The University of Gezira offers online E-learning for a master’s degree in health professional education.

In response to COVID-19, all universities and colleges in Sudan commanded their students to stay home so the government could handle the situation. Consequently, medical training has been invariably affected; however, few private universities have explored the use of online for academic activities. The effectiveness of these learning platforms in Sudan has been questioned because of poor internet connectivity, relatively expensive out-of-pocket spending on internet data bundles and electricity challenges, especially in remote rural areas. Moreover, lack of experts and limited access to the online platform are other challenges. This survey was conducted to determine the perception of medical students towards E-learning, the effect of COVID1-19 on education. Additionally, this paper highlights come of the challenges and concerns for E-learning implementation in Sudan as an example of limited-resource African setting.

## Methods

### Study types

This is a descriptive cross-sectional online survey conducted during 10–25 May 2020 among the undergraduate medical students at the Faculty of Medicine, University of Gezira (FMUG), Sudan to determine the perception of medical students regarding the E-learning, COVID-19 Pandemic, and difficulties to re-establish the educational process.

### Study population

The study population included undergraduate medical students registered at FMUG and agreed to participate in this study. Survey responses were collected anonymously.

### Study area

FMUG was established in 1975. It is situated in Wad Medani city, the capital of Gezira State. Though the Faculty of Medicine was established in 1975 the first batch of students was enrolled in 1978 and it is the second oldest medical college in Sudan. During the study period there were 1700 undergraduate medical students registered at FMUG. Currently, there are 42 medical schools in Sudan. FMUG is the first school to adopt the community-oriented, community-based, and problem-solving strategies in the country and is a pioneer in this innovative type of education all over the globe with social accountability. There has been close collaboration between the WHO and the Medical School since its inception.

### Data collection tools

A predesigned online-based questionnaire was developed by the principal investigator. The content accuracy and internal validity of the survey items were finalized with multidisciplinary input from the study investigators. It was then piloted on 10 medical students from outside the study sample and modifications were made according to the suggestions. The questionnaire was composed of 16 questions divided into three sections. All questions were labeled with serial numbers. The data collected included: Demographics (age, sex, residence, and semester), Attitude toward COVID-19, and Knowledge, experience, and attitude toward online education. The type of questions used included: Yes/No questions, four response questions in a form of strongly agree, agree, disagree, and strongly disagree (modified Likert scale) as well as other open questions regarding factors to implement starting E-learning. The questionnaire was sent to students’ email addresses and a brief informed consent stated in the opening of the electronic questionnaire. Participants were invited to share the survey link via social media platforms with other medical students in FMUG through a snowball sampling method.

### Data analysis

Data were entered and analyzed using SPSS (Statistical Package for Social Science); version 24. Categorical variables are presented as frequencies and percentages and continuous data are presented as means (standard deviation) or median values (range) depending on normality. Chi-square test was used to determine factors associated with medical students’ opinion regard education and E-learning during COVID-19 pandemic. *P* < 0.05 was considered as statistically significant.

## Results

### Characteristics of the participants

The total number of undergraduate medical students who agreed to participate in the study was 358. The mean (± SD) age was 20.4 (2.07) years and ranged between 17 and 27 years. Females represented 57.8 % of the study population. The responders were from all over Sudan; even though 57.5 % were from Gezira state and 8.4 % were from other countries. 32.1 % of subjects had laptops, and 88.5 % had smartphones which used to access the internet. The number of respondents blessed by having static internet services was 145 (40.5 %). The other detailed characteristic of the participants are shown in Table 1.

**Table 1:** Character of students (Participants) *N*=358

Variables	Frequency	%
**Gender**		
Female	207	57.8
Male	151	42.2
**Residence**		
Inside Sudan inside Gezira	199	55.6
Inside Sudan outside Gezira	129	36.0
Outside Sudan	30	8.4
Patches		
37	53	14,8
38	37	10,3
39	69	19.3
40	67	18,7
41	53	14,8
42	79	22.1
**Do you have static Internet service?**		
No	145	40.5
Yes	213	59.5
**Do you have a laptop?**		
No	115	32.1
Yes	243	67.9
**Do you have a smart phone with reasonable facility**		
No	41	11.5
Yes	317	88.5

### Perceptions and opinions of medical students towards the effect of the COVID-19 pandemic in education, cessation of education, and restart of the educational process

**Fig. 1:**
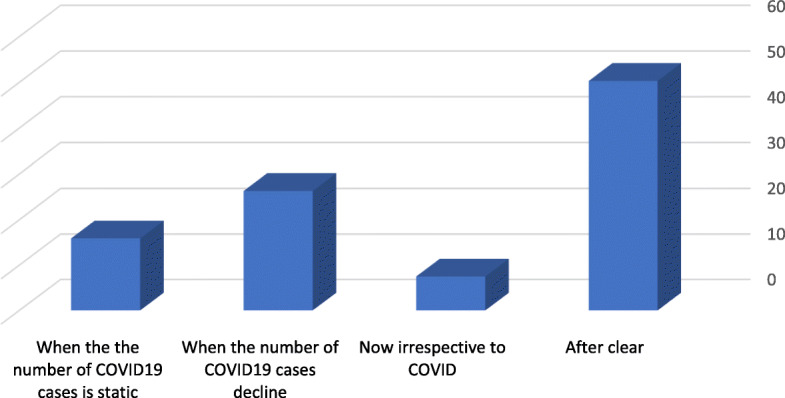
Students view regarding the suitable time to re-start learning process and open the University

The response of medical students regarding their perceptions and opinions towards the effect of the COVID-19 pandemic in education, cessation of education, and restart of the educational process are shown in Table 2. Most students (51 %) strongly agree and 31 % agree that the closure of the university is an essential decision to control the spread of the COVID-19 infection. On the other hand, 12.3 % reported that university closure is unnecessary. Approximately two-thirds (64 %) of students agreed that E-learning is the best solution during COVID-19 lockdown. The majority (69.8 %) of surveyed medical students agreed or strongly agreed that this is a high time to continuing education through E-learning. Approximately two-thirds strongly agree or agree to attend the E-learning sessions and exams during the COVID-19 pandemic. Figure 1 showed medical students’ opinion regards the suitable time to re-start campus didactic lectures, tutorials and clinical rotation. The stage of medical students (Pre-clerkship and Clerkship) and place of residence were significantly associated (*p*-value < 0.05) with their responses to the survey question “If the distant/online education is started soon, you will agree and attend the session and exams?” as shown in Table 3.

**Table 2:** Perceptions of Medical students towards the effect of the COVID-19 pandemic in Education, cessation of education, and restart of the educational process

	Strongly agree	Agree	Disagree	Strongly disagree	Mean Likert’s score
The closure of university is useful to prevent COVID-19	184 (51.4 %)	130 (36.3 %)	29 (8.1 %)	15 (4.2 %)	3.3
The worldwide closure is useful to prevent COVID-19	155 (43.3 %)	171 (47.8 %)	24 (6.7 %)	8 (2.2 %)	3.3
If the distant/online education is started soon, you will agree and attend the session and exam	117 (32.7 %)	124 (34.6 %)	64 (17.9 %)	53 (14.8 %)	2.9
This is high time Regarding the continuing education through distant / online education	74 (20.7 %)	167 (49.2 %)	74 (20.7 %)	34 (9.5 %)	2.7

**Table 3:** Relations of student's level and Residence to closure of university and start of education / E-Learning

		total	Level of Students			Residence of Students			
			Clerk (*n*=90)	Pre-clerk (*n*=268)	*P* value	Inside Gezira (n=199)	Outside Gezira (*n*=129)	Outside Sudan (*n*=30)	*P* value
**The closure of university is useful to prevent COVID**	Yes	314	84(93.3)	230(85.8)	0.065	177(87.9)	112(86)	25(83.3)	0.491
	No	44	6	38		22	17	5	
**If the distant/online education is started soon, you will agree and attend the session and exams?**	Yes	241	75(83.3)	166(61.9)	0.001	130(65.3)	84(65.1)	27(90)	0.002*
	No	117	15	102		69	45	3	
**This is high time Regarding the continuing of distant / online education**	Yes	250	74(82.2)	176(65.7)	0.02	138(69.3)	87(67.4)	25(83.3)	0.227
	No	108	16	92		61	42	5	

### Factors against the E-learning implementation in Sudan

**Fig. 2:**
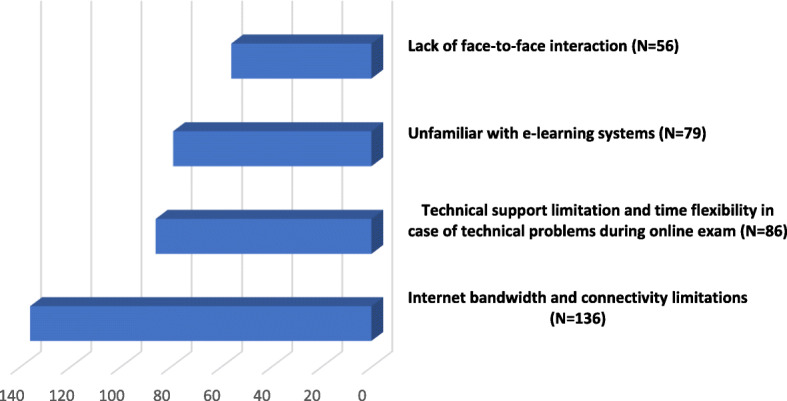
Students’ opinions (*N* = 358) regarding factors that considered being against the e. learning implementation

However, there are 4 factors considered to be against the E-learning implementation as shown in Fig. 2. More than one-third (38 %) of the student stated that good quality internet service is expensive, and the Affordable Internet services are poor in quality. Therefore, it difficult to attend live lectures or download media files. Moreover, in certain areas, due to geographical limitations, the telecommunication signal is quite hampered. 24 % reported that they were unfamiliar with E-learning systems. Approximately 40 % (*n* = 140) were concerned about technical support for online sessions and flexibility in case of technical problems during online exams. Eighty-six (24 %) students were concerned about interaction with each other and the instructors during the online session.

## Discussion

E-learning as a teaching tool of medical education can offer an effective alternative to the traditional on-site education format and help to solve the problem of shortage of health care providers and educators [6, 11, 12]. Hugenholtzet al. found that E-learning is just as effective in enhancing knowledge as lecture-based learning [13]. Moreover E-learning was found to be associated with cost reduction compared to traditional methods of education [14]. However, access to communication technologies and secure internet capacity together with poor infrastructure and institute experiences of performing E-learning are major hurdles to overcome in ensuring the success of E-learning [15, 16]. Additionally, lack of technical skills and insufficient computer skills were the barriers that can inhibit educator’s willingness or ability to engage with the development or delivery of E- learning [17, 18].

The COVID-19 outbreak has forced medical schools to suspend campus learning in order to curb the spread of the virus. Currently, medicals schools in Sudan are closed due to the COVID-19 health threat. In such situations, E-learning is the best solution that provides an online interactive learning environment for medical students without getting much affected during COVID-19 outbreak. In the developed world, many academic institutions have begun to adapt to the pandemic and are using E-learning. However,in limited-resource countries, like Sudan, shifting towards E-learning requires many adjustments to be made in order to make sure the E-learning is held in a proper manner, as best as possible.

In our study setting, FMUG undergraduate medical students have no exposure to E-learning. Recently, the Medical Education Development Center-University of Gezira offers On-line E-learning for a master’s degree in health professional education. In our limited resource setting, factors that can influence success or failure of E- learning programme are not well documented. It is known that students’ willingness and acceptance to use E-learning is a major factor in the success of e-learning system. Furthermore, a better understanding of the students’ requirements will help the decision maker to adopt E-learning successfully. Therefore, study of the perceptions of medical students at FMUG regards E-learning may help the success of the adoption of E-learning in our poor resource setting.

As far as we know, this is the first study concerning medical students’ perception twords E-learning during the COVID-19 outbreak in Sudan. A predesigned online-based questionnaire was used for data collection was developed through literature search. We strived to avoid non-response bias by using neutral wording. Additionally the content accuracy and internal validity of the survey items were finalized with multidisciplinary input from the study investigators. The survey questionnaire was also tested on 10 medical students to ensure questions were clearly articulated and the responses options are relevant. In order to assess the perception of medical students on the effect of the COVID-19 pandemic in education we used 4 point Likert scale to force the students to form an opinion i.e. no neutral opinion. We were unable to distributing the questionnaire to all medical students because the survey was conducted during COVID-19 lockdown and potential participants are hard to access. Therefore participants were invited to share the survey link via social media platforms with other medical students through a snowball sampling method. Survey data are limited by reliance on self reporting, and are potentially biased by non-responders. Our study sample was small, and these data should therefore be considered preliminary.

Compared with High income countries, the use of information and communications technology (ICT) in education programs in limited resource nations is relatively limited. Nevertheless, in recent years, there has been growing interest in the use of ICT in educational settings in developing countries. The use of ICT in undergraduate medical education in Africa lags behind that in other regions [19]. Access to technology among university students varies greatly across the African continent, so it would stand to reason that there are also disparities when it comes to accessing E-Learning tools. Technical issues, including connectivity and communications infrastructure, cost of accessing the infrastructure that is in place and lack of adequate number of competent academic staff are considered as the most significant factors in restricting E-learning in Sudan [20]. Recently, Sudan has increasingly used ICT in higher education institutions [21].

In our study, approximately two-thirds of respondents reported that good quality internet connection is too expensive for them and the affordable bandwidth is limited, which often contributed to slow speed of download and low quality of videos or visual outputs. Moreover, in remote rural areas telecommunication signal is quite hampered. The poor internet connectivity as a barrier for E-learning in medical education has been reported from another low income country context [22]. A previous study from India reported that 82 out of 201 of the planned E-learning sessions were canceled due to technical reasons (20 %) or no availability of the presenter at the host end (80 %) [23]. Therefore, in the context of low and middle income countries especially in Africa, E-learning resources should not be restricted to the Internet only and internet resources should be available in low-graphic or text‐only versions to minimise download times.

In this study, only one-third of the students have access to computers. This figure is low and comparable to studies carried out in Sub Saharan African countries [24, 25]. The majority of the surveyed medical students have smartphones with reasonable facilities. Smartphones were reported as the main mobile device used for E-learning in African higher education institutions [16]. A pervious study conducted at Central University College in Ghana showed that mobile learning enhanced collaboration between lecturers and students [26]. Moreover, smartphone E-learning applications have been effectively providing e-learning resource for resident physicians in rural areas [27]. Therefore, E-learning software that is user-friendly and easy to operate with a smartphone is needed in our setting. Just like any other technology, mobile devices have limitations especially within Africa and other developing regions. Previous studies from African countries showed that most of the students operating learning management system on mobile phones reported that using mobile devices was very slow especially in loading pages because it needed a large memory, which was lacking in most phones owned by students [28, 29].

We found 24 % of our study population being hostile to accept E-learning because they are unaware of the effectiveness of E-learning compared to the face-face teaching style and are unfamiliar with E-learning systems. Therefore, knowledge on effectiveness of E-learning among medical students is extremely important in our limited resource setting. It has been reported that the lack of face-to-face communication with lecturers and students during E-learning sessions contributed to a poor environment for professional communication and the exchange of learning experiences [14]. Similarly, we found that lack of face-to-face interaction was considered as an inhibitory factor for E-learning implementation by 15 % of our study population. Therefore, faculty administrators should develop strategies for increasing and ensuring higher levels of students’ engagement in and during E-learning.

After summarizing the response of students to the open questions, we found that 42.4 % of the respondents were worried that E-learning may need specific preparations. Further study is needed to further investigate what factors considered to make them worried about this topic. If lack of understanding of how the E-learning software runs, it means that the information technology staff must be educating/socializing in more detail to a more limited and specific group. There is also fear among our study population about the methods for online assessment and time flexibility in case of technical problems.

Medical students at the clerk level and those from outside Sudan were more likely to agree to start E-learning and attend the session and exams (*p*-value < 0.05). This could be because students from outside Sudan (Gulf countries) have access to a good quality internet connection. Further study is still needed to further investigate the critical success factors that influence E-learning acceptance among medical students.

The findings of this study were presented to the faculty assembly and decision was made to implement E-learning for some courses as a pilot project. Future study comparing students’ expectations prior to the commencement of the programme and the success of the programme is required.

The study has several limitations. The small sample size from a single medical school in central Sudan limits the generalizability of our results and the data should be interpreted with caution. Moreover, the sample may not be representative of all medical students as there is a potential for selection bias in distributing via the internet as medical students with access to the internet during the study period were more likely to participate in the study. In this study, data were collected at only one point in time (cross-sectional design) and the researcher could not manipulate the variables. Therefore, longitudinal research is required to enhance the understanding of correlation and interrelationships among variables.

## Conclusions

This study can demonstrate the views of perception regard E-learning among medical students in a limited resource setting after an emergency such as COVID-19. Most medical students have a positive perception of E-learning. However, there are many challenges considered as inhibitory factors for utilizing electronic technologies for medical education. These challenges should be systematically evaluated and that effective strategies should be developed to overcome their inhibitory effects.

## Data Availability

All data generated or analyzed during this study are included in this published article.

## Abbreviations

COVID-19: Coronavirus disease 2019
FMUG: Faculty of Medicine University of Gezira
WHO: World Health Organization
